# Hepatic autophagy is differentially regulated in periportal and pericentral zones - a general mechanism relevant for other tissues?

**DOI:** 10.1186/1478-811X-11-21

**Published:** 2013-03-26

**Authors:** Rolf Gebhardt, Paul J Coffer

**Affiliations:** 1 Institute of Biochemistry, Faculty of Medicine, University of Leipzig, Leipzig, Germany; 2 Department of Cell Biology, University Medical Center Utrecht, Utrecht, 3584CX, The Netherlands; 3 Division of Pediatrics, Wilhelmina Children’s Hospital, Utrecht, 3584CA, The Netherlands

**Keywords:** Autophagy, Essential amino acids, FOXO transcription factors, Glutamine, Hedgehog signalling, Liver, Lipophagy, Metabolic zonation, mTORC1, Wnt signalling

## Abstract

**Background:**

Liver zonation, the fact that metabolic pathways are spatially separated along the liver sinusoids, is fundamental for proper functioning of this organ. For example, glutamine synthesis from glutamate and ammonia is localized pericentrally in only 7% of the hepatocytes concentrically arranged around the central veins. Recently, we found that FOXO transcription factors lead to upregulation of glutamine synthetase expression inducing autophagy via increasing glutamine production. Since in liver this mechanism can only be functioning in the pericentral zone it remains unclear how autophagy might be regulated in the rest of liver parenchyma.

**Presentation of the hypothesis:**

We hypothesize that the regulation of autophagy by glutamine in liver is zonated. In the periportal zone, autophagy is inhibited by low intracellular glutamine but high essential amino acids, while in the pericentral zone it is stimulated by high intracellular glutamine. This zonation may be controlled by the Wnt and Hedgehog signalling pathways through reciprocal influence on the expression of amino acid transporters and metabolic enzymes in the different zones of the parenchyma.

**Testing the hypothesis:**

The hypothesis can be tested in transgenic mice with conditional hepatocyte-specific modulation of Wnt and Hedgehog signalling. Isolated periportal and pericentral hepatocyte populations allow for determining the different activities of autophagy and its regulating mechanisms in different zones of the parenchyma.

**Implications of the hypothesis:**

Zonation of the regulation of autophagy may allow adapting the extent of the proteolytic breakdown of proteins and organelles to different physiological needs in different zones of liver parenchyma. In this manner metabolic functions can be supported in one zone, for example maintenance of blood glucose levels during starvation which is a periportal issue, while simultaneously preventing cytotoxic events in the opposite zone. Likewise, lipid metabolism can be differentially influenced by uncoupling periportal lipophagy from pericentral breakdown of peroxisomes. Further implications concern the shaping of morphogen gradients along the sinusoidal axis by autophagy, and the different contribution of autophagy to the development of various different liver pathologies. The proposed dependence of the dual glutamine-dependent regulatory mechanisms of autophagy on inverse gradients of Wnt and hedgehog signalling may be relevant for other tissues in which GS is heterogeneously expressed.

## Background

Autophagy, a highly conserved homeostatic mechanism for the degradation and recycling of bulk cytoplasm, long-lived proteins and organelles [1], is triggered and modulated by a plethora of different stimuli such as nutrient deprivation and other stress signals. One such mechanisms that has recently been revealed [2] comprises a signalling cascade initiated by FOXO transcription factors that leads to transcriptional upregulation of glutamine synthetase (GS) expression, increasing enzyme activity and, therefore, glutamine production. By using different autophagic markers that label autophagosome formation, it was confirmed that FOXO-dependent activation of GS induces autophagy [2]. In the liver, this mechanism seems to be restricted to the pericentral zone for several reasons. First, GS expression is controlled by Wnt signalling [3,4] and, thus, is found in approximately 7% of the hepatocytes exclusively localized around the central vein where the gradient of Wnt signalling is culminating [3,5]. Second, FOXO3 is also predominantly localized and activated in the pericentral zone [5].

But what about the remaining 93% of the hepatocytes? Which mechanisms might dominate the control of autophagy in the majority of liver parenchyma? Up to now, there is no answer to this question. However, since early and recent measurements on the regulation of autophagy in whole liver have revealed downregulation of autophagic protein degradation by exposure to glutamine [6,7], a mechanism opposite to FOXO-mediated autophagy seems likely. It should be emphasized that this problem may hold for every tissue where expression of GS is not uniform, but cell-type specific, such as in kidney [8] or skin [9].

## Hypothesis

On this background, the following hypothesis is advanced:

1. Autophagy in liver is zonated being regulated by different, but related mechanisms in the periportal and pericentral zones of the parenchyma.

2. FOXO-mediated autophagy prevails in the pericentral zone, while the mechanism operating periportally, in the rest of liver parenchyma, may resemble the bidirectional amino acid transport mechanism suggested by Nicklin et al. [10]. This mechanism is based on glutamine as well, but here glutamine is postulated to act by facilitating the cellular uptake of essential amino acids (EAA) such as leucine which then activate mTORC1 rather than being the primary trigger of mTORC1 as in FOXO-mediated autophagy. Thus, we hypothesize that autophagy is stimulated by (high) intracellular glutamine in the pericentral zone, while it is inhibited by (high) extracellular glutamine and EAA in the periportal zone.

3. Hedgehog (Hh) and Wnt morphogen pathways according to their function as master regulators of liver metabolism [4] control the balance of autophagy in different zones of liver parenchyma. While Wnt signalling is associated with FOXO-mediated autophagy through control of GS expression [5], Hh signalling may control the suggested periportal mechanism by regulating respective amino acid transporters.

4. The glutamine-dependent mechanisms described for controlling zonation do not exclude other metabolic and hormonal signals from participating in the regulation of autophagy through influencing mTORC1 or other essential mediators. Such signals may modulate the magnitude rather than the zonation of autophagy in the given zones or cell types, a mode of action observed for most hormones and metabolic signals that regulate other zonated metabolic pathways in liver such as carbohydrate or amino acid metabolism [5,11].

## Liver glutamine metabolism and zonation of autophagy

Zonation of metabolic pathways in different areas of liver parenchyma is of considerable importance for liver function [11]. In order to understand the opposing effects of glutamine on autophagy proposed herein, some fundamental features of the zonation of glutamine and ammonia metabolism in the liver must be discussed. Glutamine is subject to intrahepatic cycling [12]: it enters the liver via the portal vein and the hepatic artery, is taken up via system N which is localized periportally [13] and degraded to ammonia and glutamate by periportal glutaminase [14]. While ammonia is mainly converted into urea by the periportal urea cycle enzymes, glutamate and the remaining ammonia are delivered to the pericentral zone and used by GS for re-synthesizing glutamine which is exported from the pericentral hepatocytes into the hepatic vein. This intrahepatic cycling plays a considerable role in determining the balance of ammonia detoxification [4,14,15].

Since periportal autophagy, according to our hypothesis, depends on external glutamine (and EAA), its activity may vary considerably in different nutritional states. In contrast, pericentral FOXO-mediated autophagy may permanently be active at a high level, because of the constantly high intracellular concentrations of glutamine in pericentral hepatocytes. The higher activity makes sense, since pericentral hepatocytes usually are exposed to more severe oxidative stress due to the predominant expression of many cytochrome P450 isozymes in this zone [16]. However, despite its potentially lower activity, the “periportal” mechanism may dominate on average, because it is at least 10-fold more abundant in the liver compared to FOXO-mediated autophagy which is restricted to the GS-positive zone (< 10% of the parenchyma). Therefore, our hypothesis provides a simple explanation for the previous findings that the average autophagic capacity in perfused liver or cultured hepatocytes is downregulated by glutamine [6,7].

## Implications

Autophagy is known to play a considerable role in liver physiology and pathology [17-19]. Zonated regulation of this process may offer not only the possibility to differently connect autophagy with anabolic and catabolic pathways which are usually inversely zonated, but also to influence these pathways in different ways. Since our hypothesis includes both, metabolic regulation through amino acids and morphogen signalling controlling the proportion of zonated functions, the implications for liver metabolism and pathology are very versatile. Some examples are discussed below.

Under well nourished conditions, amino acids (including glutamine and EAA) entering through afferent vessels are high. The suggested regulatory mechanism for periportal autophagy implies that part of the glutamine taken up is re-exported for exchange of leucine which subsequently inhibits autophagy by activating mTORC1. This may favour maintenance of mitochondria for optimally driving urea synthesis and keeping nitrogen balanced. Simultaneously, pericentral FOXO-mediated autophagy may act largely unaffected ensuring protection against increased risk of cell deterioration due to decreasing pericentral oxygen concentrations. However, if such a well nourished condition continues over time, reduced periportal autophagy may raise p62 levels compromising degradation of ubiquitine-proteasome pathway substrates [20] and eventually resulting in liver pathology.

During starvation, the opposite scenario is likely. Levels of glutamine and EAA in portal blood are quite low. Thus, little leucine may enter the periportal hepatocytes, mTORC1 remains inhibited and autophagy is activated. This mechanism may contribute to the well-known fact that the liver can maintain blood glucose and amino acid levels by sacrificing up to 40% of its protein in an early stage of starvation [7,21,22]. This process may include both, periportal and pericentral hepatocytes, since glutamine production in pericentral hepatocytes is increased due to enhanced ammonia levels. Consequently, FOXO-mediated autophagy should also be stimulated during starvation. Interestingly, repeated starvation may cause extension of the GS-positive zone [23] and, thus, may shift the balance between the two regulatory mechanisms of autophagy in favour of FOXO-mediated autophagy.

Another important issue affected by our hypothesis concerns liver lipid metabolism. Autophagy has recently been found to play an important role in lipid metabolism particularly in liver, because activation may lead to enhanced lipid degradation (also known as lipophagy [24]), while inhibition may result in a steatotic phenotype [25]. However, the situation appears much more complex. For instance, lipophagy during starvation may have a protecting function [24] by limiting the puzzling accumulation of triglycerides occurring during a 24 h fasting period [26] due to flooding the liver with free fatty acids liberated from adipose tissue. Different contributions of periportal and pericentral autophagy may explain the observed focal rather than global distribution of lipid droplets. Furthermore, independent regulation of pericentral autophagy as hypothesized herein offers the possibility for independent regulation of peroxisomal β-oxidation of fatty acids by FOXO-mediated autophagy, because peroxisomes are preferentially found in the pericentral zone. Indeed, treating fasted rats with antilipolytic drugs resulted in changes in peroxisomal rather than mitochondrial enzyme activities [27]. Notably, peroxisome distribution can be enlarged by dihydroepiandrosterone, a drug also enlarging the GS-positive zone [28] and, thus, the zone of FOXO-mediated autophagy.

The proposed dependence of the regulation of autophagy on Wnt and Hh signalling is of particular interest, since both morphogen signalling pathways can be considered as master regulators of liver zonation. This has been demonstrated for Wnt signalling which controls amino acid, ammonia and carbohydrate metabolism [3,5,29] and, via FOXO3 and glutamine synthesis, FOXO-mediated autophagy. The contribution of Hh signalling to the control of liver zonation is still hypothetical in spite of supportive data (for review see [4]). As shown in other organs, however, Hh signalling controls lipid metabolism in adipose tissue [30] and autophagy in vascular smooth muscle cells [31]. We assume similar effects to occur in liver, particularly in the periportal zone. Further evidence suggests that autophagy may regulate Wnt signalling by promoting Dishevelled degradation [32]. Taken together, these findings may imply that autophagy is not only subject to regulation by morphogens, but conversely may contribute to shaping graded morphogen action, an as yet unsolved problem in liver [6]. Given the fact that liver zonation seems to be of considerable importance for the development of distinct phenotypic classes of hepatocellular tumors [33,34], zonated regulation of autophagy may have more impact on the development of liver cancer than thought before [35].

Moreover, since GS is heterogeneously expressed in many tissues (prominent examples are kidney [8], skin [9], and small intestine [36]) matching inverse gradients of Wnt and hedgehog signalling (for small intestine see [37]), the dual glutamine-dependent opposing mechanisms described herein, may represent a more general principle for balancing bulk protein turnover by autophagy.

## Testing

The hypothesis outlined herein is testable, although functional heterogeneity of hepatocytes *in situ* is hard to approach. However, periportal and pericentral subpopulations of hepatocytes isolated by the digitonin-collagenase technique [38] may substitute and allow measuring autophagy and its regulation *in vitro* according to published techniques [2,10]. The contribution of Hh signaling in regulating autophagy could be tested in transgenic mice with conditional knockout of Smoothened (down-regulation) or of Patched (up-regulation). Conditional liver-specific regulation can be achieved by promoter systems similar to those that have been used to manipulate Wnt or TGF-beta signaling [3,29,39]. In order to evaluate zonation, periportal and pericentral subpopulations of hepatoctyes from these transgenic mice again are suitable for measuring differences in transport of amino acids, particularly glutamine, in mTORC1 activity and other known relevant functions.

## Abbreviations

EAA: Essential amino acids; FOXO: Forkhead box class O transcription factors; GS: Glutamine synthetase; Hh: Hedgehog; mTORC1: Mammalian target of rapamycin complex 1; TGF: Transforming growth factor; Wnt: Wingless-type MMTV integration site family member.

## Competing interests

The authors declare that they have no competing interests.

## Authors’ contributions

PJC participated in the design of this study and in drafting the manuscript. RG conceived of the hypothesis, and drafted the manuscript. All authors read and approved the final manuscript.
